# q2-sample-classifier: machine-learning tools for microbiome classification and regression

**DOI:** 10.21105/joss.00934

**Published:** 2018-10-23

**Authors:** Nicholas A Bokulich, Matthew R Dillon, Evan Bolyen, Benjamin D Kaehler, Gavin A Huttley, J Gregory Caporaso

**Affiliations:** 1 The Pathogen and Microbiome Institute, Northern Arizona University, Flagstaff, AZ, USA; 2 Research School of Biology, Australian National University, Canberra, Australia; 3 Department of Biological Sciences, Northern Arizona University, Flagstaff, AZ, USA

## Abstract

q2-sample-classifier is a plugin for the QIIME 2 microbiome bioinformatics platform that facilitates access, reproducibility, and interpretation of supervised learning (SL) methods for a broad audience of non-bioinformatics specialists.

Microbiome studies often aim to predict outcomes or differentiate samples based on their microbial compositions, tasks that can be efficiently performed by SL methods (Knights et al., 2011). The goal of SL is to train a machine learning model on a set of samples with known target values/class labels, and then use that model to predict the target values/class membership of additional, unlabeled samples. The ability to categorize new samples, as opposed to describing the structure of existing data, extends itself to many useful applications, e.g., the prediction of disease/susceptibility (Pasolli, Truong, Malik, Waldron, & Segata, 2016; Schubert, Sinani, & Schloss, 2015; Yazdani et al., 2016), crop productivity (Chang, Haudenshield, Bowen, & Hartman, 2017), wine chemical composition (Bokulich et al., 2016b), or sample collection site (Bokulich, Thorngate, Richardson, & Mills, 2013); the identification of mislabeled samples in microbiome data sets (Knights et al., 2011); or tracking microbiota-for-age development in children (Bokulich et al., 2016a; Subramanian et al., 2014).

We describe q2-sample-classifier, a QIIME 2 plugin to support SL tools for pattern recognition in microbiome data. This plugin provides several SL methods, automatic parameter tuning, feature selection, and various learning algorithms. The visualizations generated provide portable, shareable reports, publication-ready figures, and integrated decentralized data provenance. Additionally, integration as a QIIME 2 plugin streamlines data handling and supports the use of multiple user interfaces, including a prototype graphical user interface (q2studio), facilitating its use for non-expert users. The plugin is freely available under the BSD-3-Clause license at https://github.com/qiime2/q2-sample-classifier.

The q2-sample-classifier plugin is written in Python 3.5 and employs pandas (McKinney, 2010) and numpy (Walt, Colbert, & Varoquaux, 2011) for data manipulation, scikit-learn (Pedregosa et al., 2011) for SL and feature selection algorithms, scipy (Jones, Oliphant, Peterson, & others, 2001) for statistical testing, and matplotlib (Hunter, 2007) and seaborn (Waskom et al., 2017) for data visualization. The plugin is compatible with macOS and Linux operating systems.

The standard workflow for classification and regression in q2-feature-classifier is shown in Figure 1. All q2-sample-classifier actions accept a feature table (i.e., matrix of feature counts per sample) and sample metadata (prediction targets) as input. Feature observations for q2-sample-classifier would commonly consist of microbial counts (e.g., ampliconsequence variants, operational taxonomic units, or taxa detected by marker-gene or shotgun metagenome sequencing methods), but any observation data, such as gene, transcript, protein, or metabolite abundance could be provided as input. Input samples are shuffled and split into training and test sets at a user-defined ratio (default: 4:1) with or without stratification (equal sampling per class label; stratified by default); test samples are left out of all model training steps and are only used for final model validation.

The user can enable automatic feature selection and hyperparameter tuning, and can select the number of cross-validations to perform for each (default = 5). Feature selection is performed using cross-validated recursive feature elimination via scikit-learn’s RFECV to select the features that maximize predictive accuracy. Hyperparameter tuning is automatically performed using a cross-validated randomized parameter grid search via scikit-learn’s RandomizedSearchCV to find hyperparameter permutations (within a sensible range) that maximize accuracy.

The following scikit-learn (Pedregosa et al., 2011) SL estimators are currently implemented in q2-sample-classifier: AdaBoost (Freund & Schapire, 1997), Extra Trees (Geurts, Ernst, & Wehenkel, 2006), Gradient boosting (Friedman, 2002), and Random Forest (Breiman, 2001) ensemble classifiers and regressors; linear SVC, linear SVR, and nonlinear SVR support vector machine classifiers/regressors (Cortes & Vapnik, 1995); k-Neighbors classifiers/regressors (Altman, 1992); and Elastic Net (Zou & Hastie, 2005), Ridge (Hoerl & Kennard, 1970), and Lasso (Tibshirani, 1996) regression models.

## Figures and Tables

**Figure 1:**
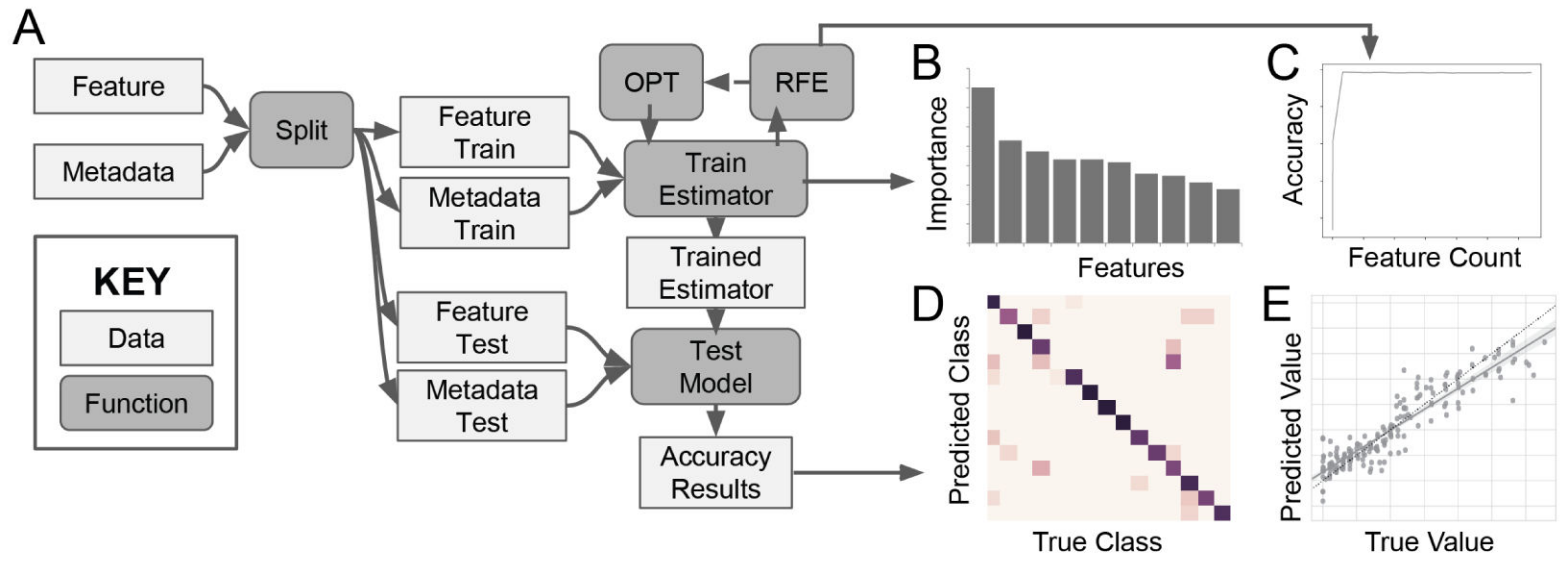
Workflow schematic (A) and output data and visualizations (B-E) for q2-feature-classifier. Data splitting, model training, and testing (A) can be accompanied by automatic hyperparameter optimization (OPT) and recursive feature elimination for feature selection (RFE). Outputs include trained estimators for re-use on additional samples, lists of feature importance (B), RFE results if RFE is enabled (C), and predictions and accuracy results, including either confusion matrix heatmaps for classification results (D) or scatter plots of true vs. predicted values for regression results (E).
